# NF-YA Overexpression in Lung Cancer: LUSC

**DOI:** 10.3390/genes10110937

**Published:** 2019-11-17

**Authors:** Eugenia Bezzecchi, Mirko Ronzio, Diletta Dolfini, Roberto Mantovani

**Affiliations:** Dipartimento di Bioscienze, Università degli Studi di Milano, Via Celoria 26, 20133 Milano, Italy; eugenia.bezzecchi@studenti.unimi.it (E.B.); mirko.ronzio@unimi.it (M.R.); diletta.dolfini@unimi.it (D.D.)

**Keywords:** lung cancer, LUSC, transcription factors, TCGA, CCAAT box, NF-YA, alternative splicing

## Abstract

The CCAAT box is recognized by the trimeric transcription factor NF-Y, whose NF-YA subunit is present in two major splicing isoforms, NF-YAl (“long”) and NF-YAs (“short”). Little is known about the expression levels of NF-Y subunits in tumors, and nothing in lung cancer. By interrogating RNA-seq TCGA and GEO datasets, we found that, unlike NF-YB/NF-YC, NF-YAs is overexpressed in lung squamous cell carcinomas (LUSC). The ratio of the two isoforms changes from normal to cancer cells, with NF-YAs becoming predominant in the latter. NF-YA increased expression correlates with common proliferation markers. We partitioned all 501 TCGA LUSC tumors in the four molecular cohorts and verified that NF-YAs is similarly overexpressed. We analyzed global and subtype-specific RNA-seq data and found that CCAAT is the most abundant DNA matrix in promoters of genes overexpressed in all subtypes. Enriched Gene Ontology terms are *cell-cycle* and *signaling*. Survival curves indicate a worse clinical outcome for patients with increasing global amounts of NF-YA; same with hazard ratios with very high and, surprisingly, very low NF-YAs/NF-YAl ratios. We then analyzed gene expression in this latter cohort and identified a different, pro-migration signature devoid of CCAAT. We conclude that overexpression of the NF-Y regulatory subunit in LUSC has the scope of increasing CCAAT-dependent, proliferative (NF-YAs^high^) or CCAAT-less, pro-migration (NF-YAl^high^) genes. The data further reinstate the importance of analysis of single isoforms of TFs involved in tumor development.

## 1. Introduction

The process of cellular transformation entails profound changes in gene expression. As a consequence of genetic and/or epigenetic changes, cancer cells are characterized by alteration in their transcriptomes, compared to normal counterparts. To understand the logic behind these changes, huge efforts have been made to characterize transcription profilings of cancer cells. In general, transcription is dictated by the precise and often synergistic binding of transcription factors (TFs) to regulatory regions (promoters and enhancers) of genes [1]. Indeed, alteration of the structure and/or expression levels of many TFs leads to tumorigenesis through profound changes in gene expression. 

NF-Y is a TF binding with high specificity to the CCAAT box, an important DNA element found in promoters and enhancers. It is a heterotrimer formed by the sequence-specific NF-YA and the histone fold domain (HFD) dimer NF-YB/NF-YC [2]. NF-YA, at the N-terminal, and NF-YC, at the C-terminal, harbor Gln-rich trans-activation domains (TAD). Both are involved in alternative splicing events, generating two major isoforms of NF-YA (differing in 28 amino acids) and several isoforms of NF-YC [3,4]. These isoforms are expressed at various levels, with no immediately obvious logic, in different tissues. 

NF-Y genes are neither frequently mutated nor amplified in human cancers, yet the trimer appears to play a role in tumor development, or progression [5]. First, microarray profilings and RNA-seq analysis found “signatures” genes for some types of cancers: TFBSs (Transcription Factor Binding Sites) searches identified the CCAAT box as specifically abundant [6,7,8,9,10]. CCAAT is present in promoters at a relatively precise location, −60/−100 from the TSS, and it is usually crucial for high-level expression of genes [11]. Thus, it appears that “cancer“ genes rely on the CCAAT box to be activated in tumors. Second, some experiments point at HFD subunits as direct “driver” oncogenes in specific types of cancers: NF-YB in diffuse large B-cell lymphoma (DLBCL), an aggressive evolution of follicular lymphoma [12]; NF-YC in choroid plexus carcinomas, as suggested by mouse/human synthenic screenings and functional analysis [13]. Third, systematic analysis of TFs genomic locations performed in ENCODE ChIP-seq datasets identified NF-Y intersecting with known oncogenic TFs, E2Fs, FOS, MYC [14,15].

NF-Y has been originally tagged as a “ubiquitous” TF, whose levels and DNA-binding activity vary little in different, mostly tumorigenic, cell lines. The inactivation of NF-YA by RNAi leads to cell-cycle arrest and apoptosis in different cellular contexts, and no cell line has ever been described lacking NF-Y activity. On the other hand, our knowledge about the actual expression levels of NF-Y subunits in human tumors is still at a rudimentary stage: elevated expression of NF-YA was reported in cohorts of epithelial ovarian cancer [16,17], triple negative breast cancers [18], gastric cancer [19,20], and of the NF-YC subunit in gliomas and colon adenocarcinomas [21,22]. We recently started to interrogate, both in quantitative and qualitative way, the large RNA-seq datasets of TCGA and, surprisingly, found that NF-YA is overexpressed in most tumors of epithelial origin. 

Lung cancer is a leading cause of death in industrialized countries, with clear ties to epigenetic cues [23,24]. The major type of lung cancer is non-small cell carcinoma (NSCLC) which represents 80/90%; NSCLC, in turn, are further classified in two distinct types, squamous cell carcinomas (LUSC) and adenocarcinomas (LUAD), according to clinical, histological, genetic and molecular aspects. Specifically, LUSC has been further partitioned by virtue of gene expression signatures in four subtypes: basal, classical, primitive and secretory [25]. In general, nothing is known about expression levels of NF-Y subunits in lung cancers, nor on regulation of NF-Y-dependent cancer genes in LUSC.

Here, we report on the systematic interrogation of NF-Y subunits levels in lung squamous cell carcinomas RNA-seq datasets of the TCGA consortium and of an independent study performed in Sweden [26,27].

## 2. Materials and Methods

### 2.1. RNA-seq Datasets

All available TCGA data on LUSC were obtained from the http://firebrowse.org/ web page: as of January 2019, there were RNA-seq data on 552 LUSC samples, including 501 primary tumor samples and 51 non-tumor (normal) lung tissues. Of the 198 NSCLC included in the GSE81089 dataset, we first partitioned LUSC and LUAD samples by performing Pearson correlation of each samples with a 42 genes classifier previously validated [28]. This allowed the identification of 78 LUSC samples. Samples with correlation values ranging from −0.17 and 0.17 were not further classified and were not considered while values above 0.17 are predicted to be LUAD while values below −0.17 are predicted to be LUSC.

### 2.2. Classification of All TCGA LUSC Tumors

We extended the partitioning of all LUSC TCGA tumors in subtypes. The 207 genes previously validated as signatures for the four subtypes were used for this task [25]; each gene was median centered on all 501 LUSC samples and Pearson correlations were calculated between the predictor centroids and TCGA samples. A TCGA tumor’s subtype prediction was given by the centroid with the largest correlation value.

### 2.3. Global Gene Expression Analysis

Differentially Expressed Genes (DEG) analysis of RNA-seq data was performed using R package DESeq2 [29]. The tumor versus normal (T/N) expression fold change (FC) denotes upregulation or downregulation according to the FC value. Log_2_FC, and the corresponding false discovery rate (FDR) were all reported by the R package. FDR < 0.01 and |log_2_FC| > 2 were set as inclusion criteria for DEG selection in tumor/subtype versus normal samples. 

To calculate DEG in tumors with low or high NF-YAs/NF-YAl ratios, we used FDR < 0.01 and |log_2_FC| > 1. Samples with values within the 1st quartile were considered as low ratio, those within the 2nd to 3rd quartile were considered as intermediate ratio, while samples of the 4th quartile were considered as high ratio. The above mentioned samples had clinical data available.

For NF-YA expression analysis in microarray data of LUSC subtypes [25], we used the GEO2R tool available on NCBI website, comparing each subtype with all other mRNA samples.

### 2.4. Gene Ontology and Pathway Enrichment Analysis

For pathway enrichment analysis we employed KOBAS 3.0 (http://kobas.cbi.pku.edu.cn/anno_iden.php) using the ENTREZ gene IDs. 

### 2.5. Transcription Factor Binding Site (TFBS) and De Novo Motif Discovery

To detect over-represented TFBSs, we run Pscan on the Pscan web interface [30], selecting promoter region of −450 +50 nucleotides from the transcription start site and using Jaspar_2016 descriptors. To identify novel motifs de novo we used Weeder 2.0 [31], providing as input the promoter sequences (-450 +50 nucleotides from the transcription start site) from co-regulated genes. To calculate the statistical significance, we loaded the matrices obtained from Weeder 2.0 into Pscan together with the list of enriched genes, and we obtained the Bonferroni-corrected *z*-test *p* values. The position weight logo obtained were transformed from the Weeder 2.0 matrix output with seqLogo in R [32]. 

### 2.6. Analysis of Clinical Data

We retrieved clinical data relative to the TCGA LUSC samples, including Progression Free Interval (PFI) time records of 318 patients, from the https://nborcherding.shinyapps.io/TRGAted/ web page [33]. Survival analysis was performed according to the Kaplan-Meier analysis and log-rank test [34]. Cox proportional hazard modelling of ratio and covariates was calculated to determine their independent impact on patient’s survival and to estimate the corresponding hazard ratio setting either low global NF-YA expression or intermediate ratio (NF-YAs/NF-YAl) as reference values. 

### 2.7. Statistical Analysis

All data analyses were performed in the R programming environment (version 3.2.5), using several packages (*ggplot2*, *ggsignif*, *reshape*, *ggpubr*, *clinfun*, *heatmap.plus*). Single comparisons between two groups were performed with the Wilcoxon rank sum test, while we calculated the Jonckheere’s Trend test to verify statistically significant trends between two genes of interest in a pre-ranked dataset.

## 3. Results

### 3.1. NF-YA is Overexpressed in Lung Tumors

**Figure 1:**
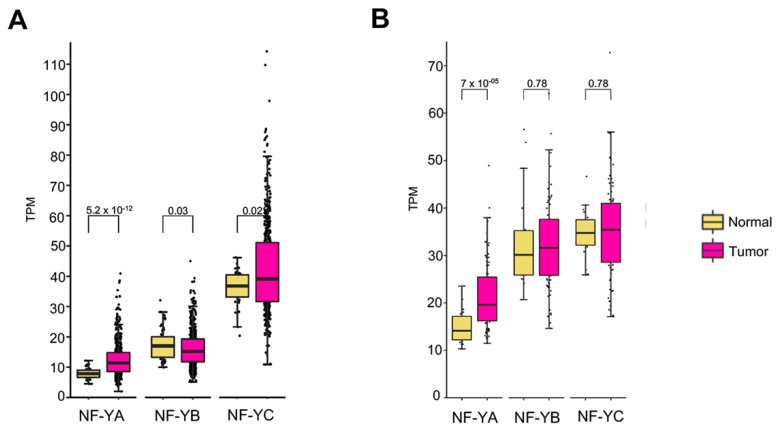
NF-YA is overexpressed in LUSC. (**A**) Box plots represent the expression levels of NF-Y subunits at gene level in the TCGA-LUSC RNA-seq dataset, measured in TPMs. (**B**) Expression of NF-Y subunits at gene level across the LUSC tumors of the GSE81089 dataset. *P* values are calculated using a Wilcoxon signed-rank test.

We used Firebrowse (http://firebrowse.org/viewGene.html) to gather a bird’s eye view on the levels of NF-Y subunits in 18 tumor types analyzed by TCGA: NF-YA, not NF-YB/NF-YC, is elevated in the majority of tumors of epithelial origin [35]. Specifically, LUSC showed high statistical significance. We thus decided to analyze systematically the LUSC dataset of TCGA [26]: quantification of RNA-seq data indicates that the levels of NF-YA are indeed increased in cancer samples compared to normal controls (*p* value 10^−12^); instead, NF-YB shows comparable levels in tumors and normal cells (Figure 1A). NF-YC also shows increased levels, albeit with modest statistical significance (*p* value 10^−2^). Note that the global levels of HFDs mRNAs, particularly NF-YC, are higher than those of NF-YA.

To confirm the TCGA findings, we interrogated another dataset GSE81089, derived mostly from a Swedish cohort of patients [27]: to the best of our knowledge, this is the largest, 198 samples, non-TCGA study reporting on RNA-seq of NSCLC. This classification contains two major types of lung cancers, LUSC and LUAD: therefore, it was first necessary to partition samples in these two types, by using a gene expression signature previously described [28]. Correlation plot and Principal Component Analysis (PCA) show substantial separation of the two groups (Figure S1A,B); the heatmap of signature genes previously identified confirms the partitioning (Figure S1C). This procedure allowed the identification of 78 LUSC tumors in this dataset. The complete list of LUAD and LUSC samples are in Table S1. We then focused on LUSC samples and assessed mRNA levels of the three NF-Y subunits, as done with the TCGA data: NF-YA is substantially increased in tumors (*p* value 10^−5^), unlike NF-YB and NF-YC (Figure 1B). These latter results confirm NF-YA overexpression, as well as normal expression of the HFD subunits, in LUSC.

### 3.2. Splicing Isoforms of NF-YA Are Differentially Regulated in LUSC

**Figure 2:**
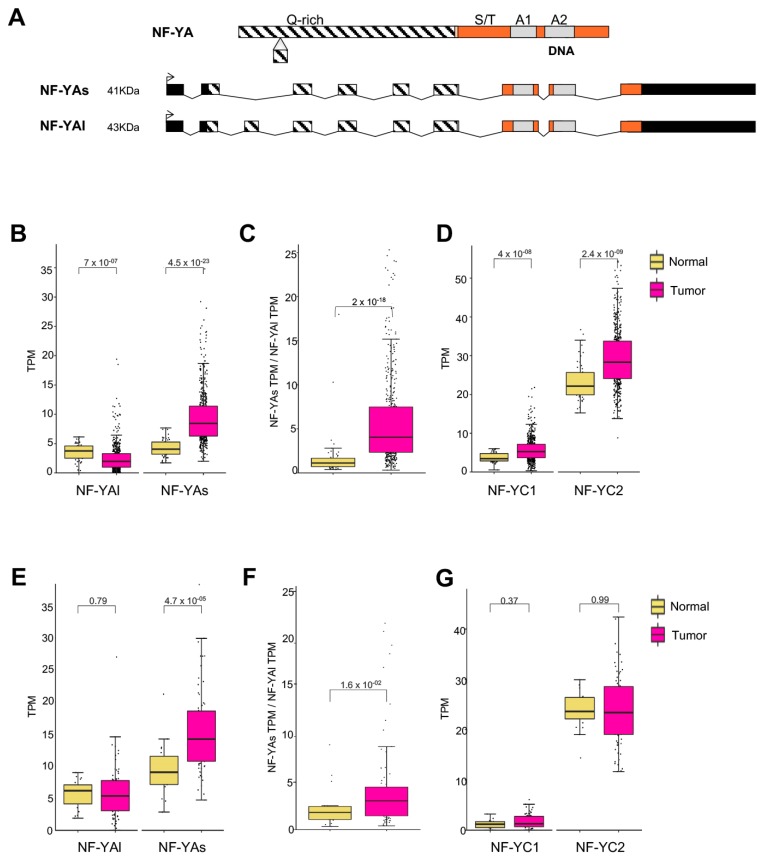
NF-YA short isoform is overexpressed. (**A**) Scheme of the NF-YA exons, with the NF-YAl and NF-YAs isoforms indicated. Q-rich, trans activation domain. S/T, serine/threonine-rich domain. A1, helix 1, subunits-interaction domain. A2, helix 2, DNA-binding domain. (**B**) Box plots of expression levels of NF-YAs and NF-YAl in the TCGA dataset. (**C**) Relative ratios of NF-YAs/NF-YAl. (**D**) Expression levels of NF-YC isoforms in TCGA LUSC. NF-YC1, 50 kD isoform. NF-YC2 37 kD isoform. (**E**) Expression levels of NF-YAs and NF-YAl in TCGA and GSE81089. (**F**) Relative ratios of NF-YAs/NF-YAl. (**G**) Expression levels of NF-YC isoforms in TCGA and GSE81089.

NF-YA and NF-YC genes are involved in alternative splicing, generating two major NF-YA (Figure 2A) and multiple NF-YC (Figure S2) isoforms. We analyzed them individually in LUSC, first in the TCGA dataset. Figure 2B shows that in normal cells there are balanced levels of the two NF-YA isoforms; the “short” (NF-YAs) is substantially increased (*p* value 10^−23^), and NF-YAl decreased (*p* value 10^−7^), in tumor samples. As a result, a robust change in the ratio of isoforms levels is produced (Figure 2C). As for NF-YC, the predominant 37 kD and 50 kD isoforms are concomitantly increased (*p* value 10^−8/9^, Figure 2D).

We then turned to the GSE81089 data: NF-YAs was statistically increased (*p* value 10^−5^, Figure 2E), whereas NF-YAl and the NF-YC isoforms are overall similar in normal and tumor samples (Figure 2E,G). Largely because of the NF-YAs increase, the NF-YAs/NF-YAl ratio is indeed increased in tumors (Figure 2F). Although differences are scored between the two datasets, mostly regarding the degree of NF-YAl decrease in tumors, the data concur that NF-YAs levels are increased in LUSC tumors. We conclude that there is an isoform switch in NF-YA isoforms in LUSC, from normal to tumor cells. 

### 3.3. NF-YA Levels Correlate with Proliferative Markers

**Figure 3:**
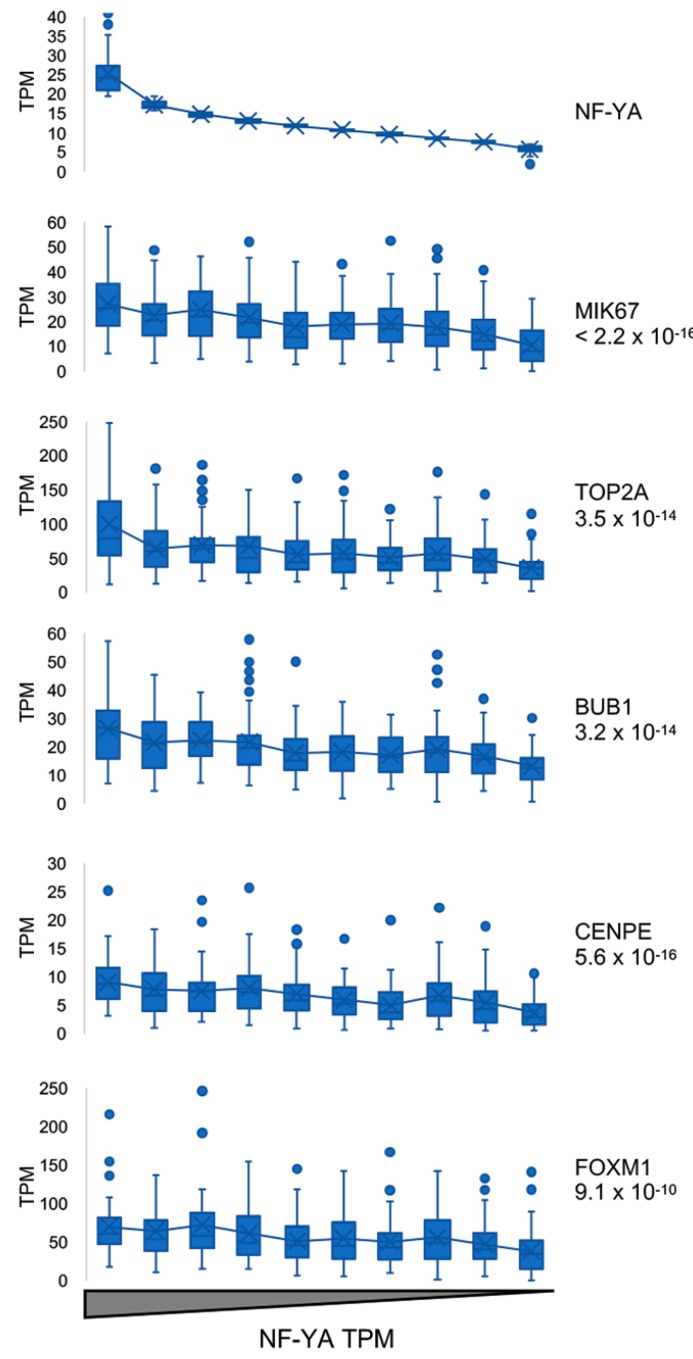
TCGA-LUSC samples ranked based on NF-YA expression. Upper panel, separation of LUSC samples in ten bins of NF-YA expression levels, according to TPM values. Lower panels, expression of Ki67 (MIK67), TOP2A, BUB1, CENPE and FOXM1.

To correlate the levels of NF-YA expression to proliferation of lung tumor cells, the 501 TCGA tumors were binned according 10 different NF-YA expression levels, from high to low (Figure 3, upper panel). Thereafter, we measured expression of five genes commonly considered as proliferative markers, Ki67, TOPO IIa, BUB1, CENP2, FOXM1, including in lung cancers [36,37,38,39]; in the 10 NF-YA cohorts, all showed progressively decreasing levels from high-to-low NF-YA expressing tumors (Figure 3). The calculated statistical significance for each gene is high (*p* values 10^−10/16^). We conclude that elevated expression of NF-YA correlates with that of a number of proliferative marker genes in LUSC.

### 3.4. NF-Y Sites are Enriched in Promoters of Genes Overexpressed in LUSC

**Figure 4:**
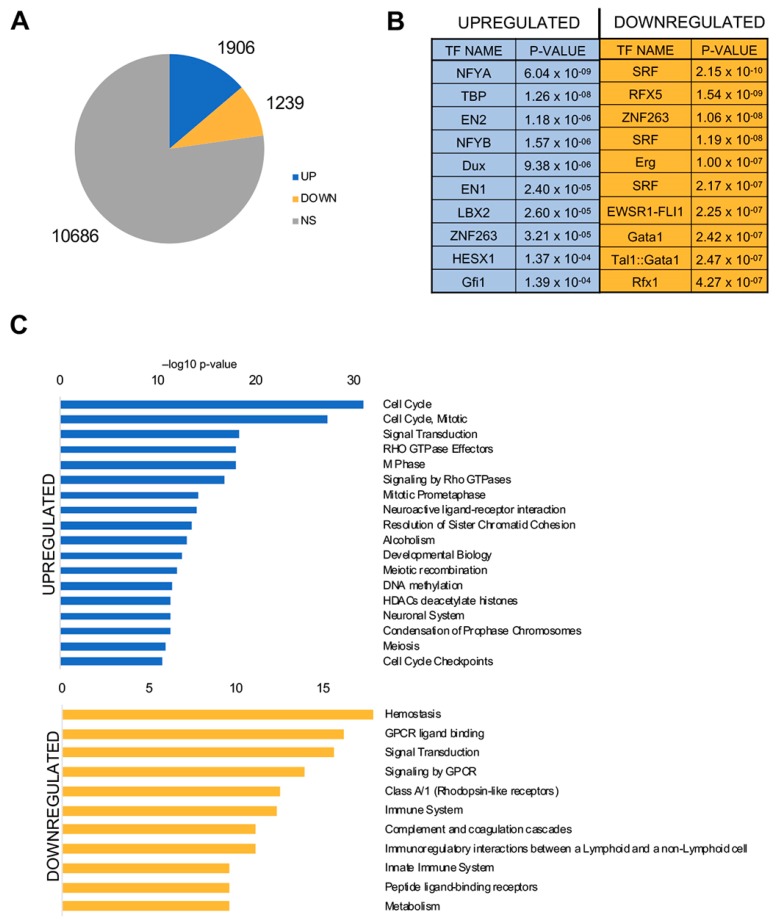
Gene expression analysis of the whole set of 501 LUSC TCGA tumors. (**A**) Up- and down-regulated genes in tumors versus normal lung tissues. (**B**) Pscan analysis of enriched TFBS in promoters (–450/+50 bps from the TSS) of up- (left panel) and down-regulated (right panel) genes in tumors. (**C**) Reactome pathways enriched in commonly upregulated genes (upper panel) and down-regulated genes (lower panel) listed according to their *p* value. The list is obtained using KOBAS.

We analyzed global RNA-seq gene expression in the 501 LUSC TCGA tumors, compared to normal tissues present in the datasets. The lists of Differentially Expressed Genes (DEG) using a |log_2_ FC| > 2, FDR < 0.01 threshold, are in Table S2. 1905 genes are overexpressed and 1238 are down-regulated, the majority, 77%, are classified as unchanged (Figure 4A). We then analyzed promoter sequences, from −450 to +50 from the TSS, of DEG with Pscan [30], an algorithm identifying enriched DNA matrices present in TFs databases (JASPAR). Figure 4B (left panel) shows that NF-Y is the most enriched matrix in up-regulated promoters. Note that NF-YA (first) and NF-YB (fourth) are essentially identical CCAAT matrices. In promoters of down-regulated genes, instead, matrices of SRF, RFX and zinc-fingers TFs, such as ZNF263 and GATA, predominate, but CCAAT is absent (Figure 4B, Right Panel). 

We then pursued analysis of Gene Ontology (GO) terms present in DEG genes, using the KOBAS software: we show enrichment of *cell-cycle*, particularly G2/M annotations such as *mitosis*, *M phase*, *prometaphase*, *chromosome condensation*, and *signaling*, notably *Rho GTPases* (Figure 4C). In down-regulated genes, we found a more variegated set of terms, stemming from *signaling* to *immunity*, *coagulation* and *metabolism*. We conclude that CCAAT boxes predominate in promoters of genes overexpressed in LUSC, mostly related to cell-cycle and signaling functions. 

### 3.5. Classification of All TCGA LUSC Tumors in Subtypes

**Figure 5:**
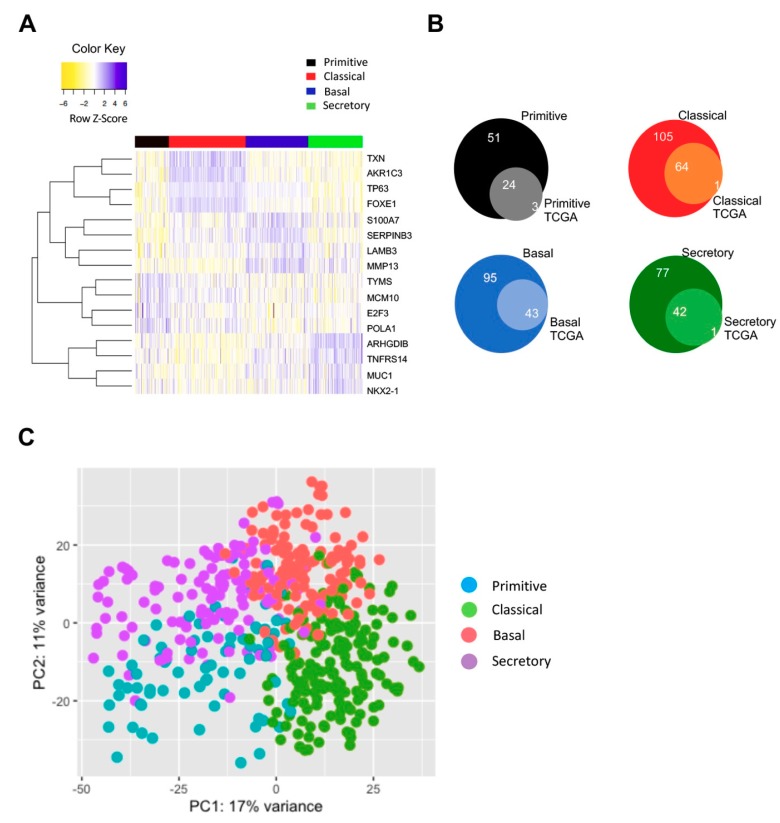
Partitioning of all LUSC TCGA tumors in four molecular subtypes. (**A**) Heatmap of 16 signature genes for each of the four subtypes of LUSC. (**B**) Venn diagrams of the four LUSC subtypes: smaller circle, previous TCGA classification of 178 tumors. Larger circles, complete classification of all 501 LUSC tumors in TCGA. (**C**) PCA (principal component analysis) of the four LUSC subtypes according to new classification.

LUSC tumors are classified in basal, classical, primitive and secretory, according to molecular criteria: the global NF-YA overexpression observed above could be limited to one (or more) of the subtypes. In TCGA, subtypes analysis was previously performed on a third (178) of the 501 available tumor samples [26]. We figured that it could be challenging to gain statistically significant results on NF-YA expression for primitive tumors, for which only 27 samples were available from TCGA. Hence, we decided to extend the subtypes classification to all 501 tumors with RNA-seq data. To do so, we took advantage of a 207 genes signature originally described by Wilkerson et al. [25], and subsequently used by TCGA. Figure 5A shows the heatmap of expression levels of 16 representative genes in LUSC samples, showing the expected partitioning. Figure S3 shows box plots of expression of genes hallmarks for each subtype: MCM10 and TYMS (primitive), TP63 and TXN (classical), ARHGDIB and TNFSRF14 (secretory), S100A7 and MMP13 (basal). Venn diagrams of the previous and new, complete classification is shown in Figure 5B; note that the relative proportions are essentially identical: basal are now 138, Primitive 75, secretory 119 and classical 169. Finally, Principal Component Analysis (PCA) of all tumor samples shows robust clustering of the subgroups (Figure 5C). The complete list with all TCGA LUSC tumors partitioned according to the four subtypes is in Table S3.

### 3.6. Expression of NF-YA Isoforms in LUSC Subtypes

**Figure 6:**
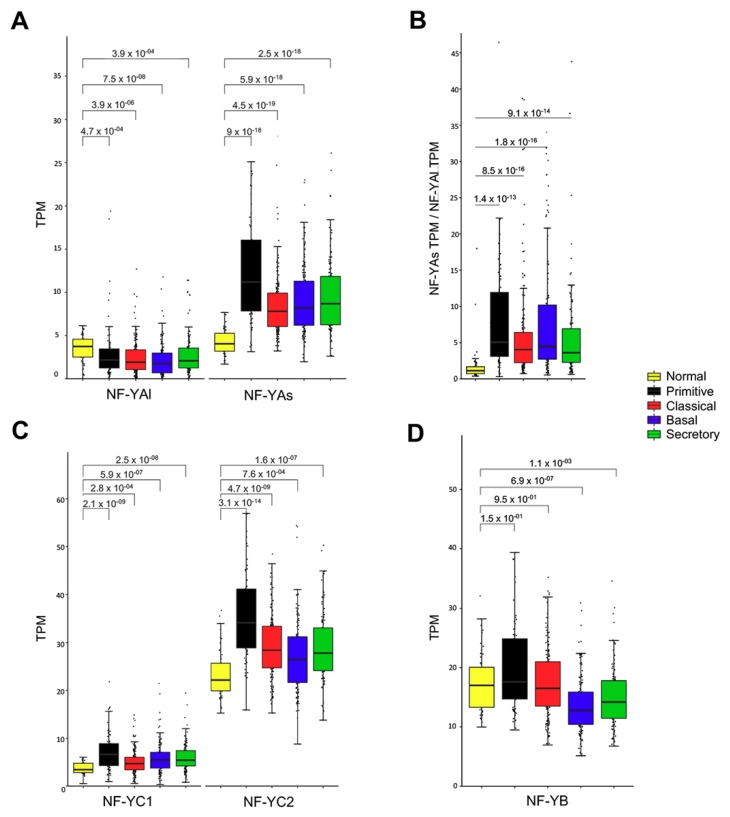
NF-YA is overexpressed in all LUSC subtypes. (**A**) Box plots represent the expression of NF-YA isoforms at gene level in the four LUSC subgroups, measured in TPM. (**B**) NF-YAs/NF-YAl ratios in the four subtypes. (**C**) Box plots represent the expression of NF-YC isoforms at gene level in the four LUSC subgroups. NF-YC1, 50 kD isoform. NF-YC2 37 kD isoform. (**D**) Box plots represent the expression of NF-YB at gene level in the four LUSC subgroups. *P* values are calculated using a Wilcoxon signed-rank test.

With the complete list of LUSC subtypes on hand, we compared the levels of the three subunits in the four subtypes with normal lung tissues. First, we compared the global levels of NF-YA (both isoforms) in the four LUSC subtypes, as found in microarray datasets [25]: primitive tumors showed the highest expression and classical the lowest (Figure S4A). Note that normal counterparts are absent in these data. We then moved to TCGA LUSC RNA-seq datasets: the same trend of NF-YA levels (both isoforms) is observed in the same subtypes as above (Figure S4B). As for the specific levels of the two isoforms, NF-YAl drops significantly in Classical and Basal (Figure 6A), NF-YAs increases in all (*p* value 10^−18/19^). As a consequence, the NF-YAs/NF-YAl ratio raises substantially in all tumors (Figure 6B). As for NF-YC, there is a generalized increase of the 37 kD, particularly in primitive (Figure 6C), indicating that the global increase observed in this subtype is essentially due to this NF-YC isoform: in fact, the NF-YC 50 kD isoform is lowly expressed. NF-YB is either not changed, or modestly decreased, in basal tumors (Figure 6D). In conclusion, the data concur that overexpression of NF-YA is widespread and not restricted to a specific subtype, and changes in HFD subunits are overall modest.

### 3.7. Genes Overexpressed in All LUSC Subtypes Have CCAAT in Promoters

**Figure 7:**
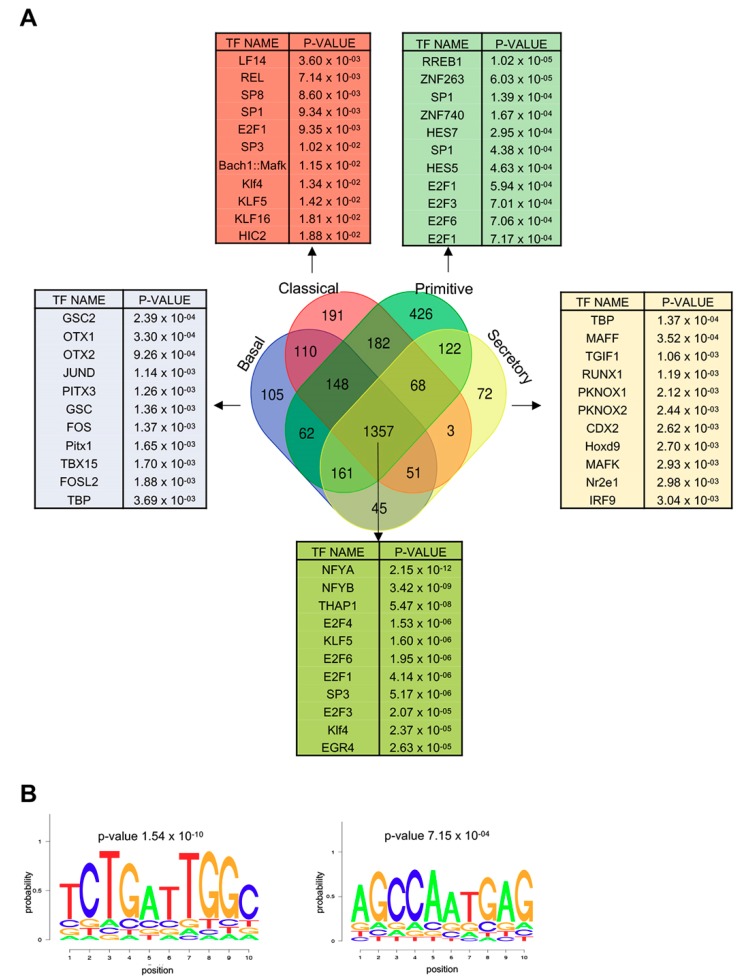
Gene expression analysis of the four LUSC subgroups. (**A**) Venn diagram of the genes overexpressed in the four subgroups of LUSC. Pscan analysis of the TFBS enriched in signatures of overexpressed genes: basal (105 genes), classical (191), primitive (426), secretory (72). At the bottom, Pscan analysis of the core group (1357) of genes overexpressed in all subgroups. (**B**) Weeder analysis of de novo motif identification of sites enriched in promoters of the 1357 common over-expressed genes. *P* values are calculated using a Wilcoxon signed-rank z-test with Bonferroni correction.

We proceeded with RNA-seq gene expression analysis of the four partitioned subtypes, with the same threshold criteria used above. The lists of DEG genes are in Table S4. As for up-regulated genes, we found subtype-specific signatures of 105 genes in basal, 191 in classical, 426 in primitive and 72 in secretory tumors (Figure 7A). Pairwise overlaps ranged from negligible (secretory/classical 3 genes) to relatively modest (classical/primitive 182 genes). Note that the vast majority of genes (1357) shows overexpression in all subtypes. We searched TFBS in the promoters of these commonly overexpressed genes with Pscan: we found a very robust enrichment of NF-Y sites (*p* values 10^−9/12^), whose matrices are at the top of the list, together with different flavors of E2Fs and Sp1/KLFs DNA motifs (Figure 7A). Remarkably, NF-Y matrices are absent from signatures of the specific subtypes (Figure 7A). To confirm these findings, the commonly overexpressed genes were run on Weeder, an algorithm identifying *de novo* DNA matrices [31]. Figure 7B shows retrieval of CCAAT as the only statistically enriched DNA site. Figure S5 shows that the most statistically enriched categories in genes commonly overexpressed are *cell-cycle* and *signaling*, while gene signatures of individual subtype have other, more specific terms (not shown).

When the same exercise was done on the subtype specific cohorts, different groups of TFBSs emerged: HOX and bZip (OTX, Jun/FOS) in basal and secretory, zinc fingers (Sp1/3/8 and KLFs) in Classical, E2Fs and zinc fingers in primitive (Figure 7A). The lower statistical significance is to be expected, given the fewer samples. 

Finally, we performed the same type of analysis in down-regulated genes (Figure S6: in the DEG common to the four subtypes (697) no CCAAT matrix was detected, but rather the SRF, GATA, RFX5 and FOXO sites, indeed reminiscent of those of Figure 4B. We conclude that genes overexpressed in all subtypes of LUSC have proliferative signatures, and their promoters harbor CCAAT boxes as well as sites of other pro-growth TFs.

### 3.8. Clinical Outcomes of NF-YA Isoforms Overexpression

**Figure 8:**
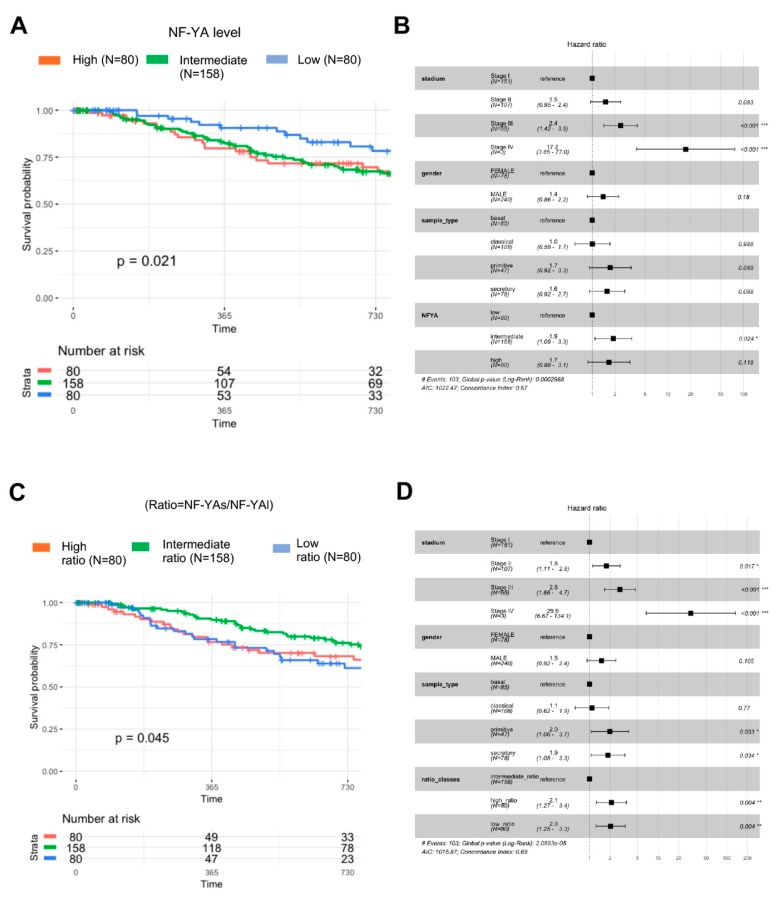
Clinical outcome of LUSC tumors with different NF-YAs/NF-YAl ratio. (**A**) Progression-free-interval curves of survival probability of the 318 LUSC tumors with available clinical data, stratified according to quartiles of global NF-YA levels (intermediate, high and low). (**B**) Hazard ratios of the three cohorts as above, according to Stage (I–IV), gender, sample type (basal set as reference) and global NF-YA levels. (**C**) Same as A, except that data were stratified according to quartiles of NF-YAs/NF-YAl ratios (intermediate, high and low). (**D**) Hazard ratios of the sample cohorts partitioned according to Stage (I–IV), gender, sample type (basal set as reference) and NF-YAs/NF-YAl ratios. *P* values are calculated using a Cox proportional hazards regression analysis (See Materials and Methods). * *p* value < 0.05, ** *p* value < 0.01, *** *p* value < 0.0001.

To investigate the potential impact on the clinical outcome of the disease, we first correlated the global levels of NF-YA with patient survival, by analyzing Progression Free Intervals (PFI), a measure that best predicts clinical outcomes [40]. We classified all tumors for which clinical features are available (318) in three cohorts: first quartile in the high levels, last quartile in the low, and the remaining grouped in the intermediate. The curves of Figure 8A show a significant drop of PFI in patients with high and intermediate NF-YA levels. We calculated the hazard ratio in the three bins of NF-YA levels: Stage II and Stage IV are predictably skewed toward highest ratio (Figure 8B). None of the other parameters, with the exception of intermediate NF-YA levels, showed a statistically significant change.

Next, we repeated the analysis based on NF-YAs/NF-YAl ratios: the first quartile with NF-YAl^high^, the last quartile with NF-YAs^high^ and the two middle quartiles having intermediate levels of the isoforms. The results of Figure 8C shows a drop in PFI with high or low NF-YAs/NF-YAl ratios compared to tumors with intermediate ratios (*p* value 0.045). 

As for hazard ratio analysis, the results are shown in Figure 8D: hazard ratios are indeed shifted in tumors with high (2.1, *p* value 0.004) and low (2, *p* value 0.004) NF-YAs/NF-YAl ratio; in secretory and primitive (2 and 1.9, *p* values 0.033), but not in the other subtypes. It progressively increases from the reference Stage I to 1.8 (Stage II), 2.2 (Stage III) and 29.9 in the few (*n* = 3) Stage IV patients. We conclude that the relative levels of the two NF-YA isoforms have an impact on the clinical outcome of LUSC: surprisingly, worst prognosis is observed not only in patients with high NF-YAs/NF-YAl ratios, but equally in those with very low.

### 3.9. LUSC Tumors with Low NF-YAs/NF-YAl Ratio Have a Distinct Gene Signature

**Figure 9:**
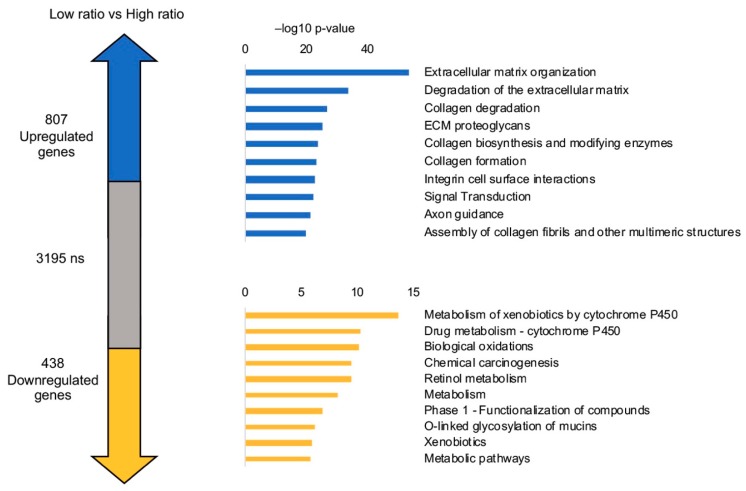
DEG analysis of 80 tumors with low NF-YAs/NF-YAl ratio. The number of up- and down-regulated genes in the comparison between tumors with low and high NF-YAs/NF-YAl ratios, as in Figure 8. Right panels, Reactome pathways enriched in up-regulated (upper panel) and down-regulated (lower panel) genes in low vs. high NF-YAs/NF-YAl ratios, listed according to their p-value. The list is obtained using KOBAS.

The clinical data shown above spurred us to further investigate gene expression of the cohort of 80 tumors with low NF-YAs/NF-YAl ratios, most of which have relatively high levels of NF-YAl. We first compared DEG -|log_2_FC| >1, FDR < 0.01- in low and high NF-YAs/NF-YAl ratios with intermediate ones (not shown) and then proceeded to directly compare low to high ratios. There are 807 up-regulated and 438 down-regulated genes in low vs. high ratios cohorts (Figure 9): GO terms in down-regulated genes are mostly associated with metabolism (xenobiotics, drugs, retinol); this is in keeping with the general gene expression analysis of LUSC tumors shown in Figure 4 and Figure 7, despite the lack of the *cell-cycle* term. Interestingly, tumors with low ratios, those generally NF-YAl^high^, show enrichment of new GO terms, linked to extracellular matrix, collagen, integrin. As the second word is specifically *degradation of the extracellular matrix*, they can be collectively associated to increased cell migration features (Figure 9). In the promoter of this cohort of up-regulated genes, Pscan analysis does not detect any enrichment of NF-Y binding sites. We conclude that the sub-group of LUSC tumors with worst prognosis, singled-out because of their comparatively higher levels of NF-YAl, is marked by high expression of CCAAT-less pro-migration genes. 

## 4. Discussion

Nothing was previously known about NF-Y in lung squamous cells carcinomas. In addition to finding an abundance of CCAAT in promoters of “cancer” genes as in other tumor types, three new findings are reported: (i) overexpression of NF-YA, notably the “short” isoform, correlating with known proliferation markers; (ii) identification of a distinct cohort of tumors with higher NF-YAl levels and a CCAAT-less pro-migration signature; (iii) negative clinical outcome of tumors with global increase of NF-YA levels as well as an opposite spectra, with low, and high, NF-YAs/NF-YAl isoforms ratios.

Abundance of CCAAT boxes in promoters of genes overexpressed in cancer was reported in microarray profiling experiments, culminating with an unbiased search for TFBSs in the vast Oncomine microarray dataset, which found the NF-Y matrix as one of the most represented [6]. On the other hand, CCAAT was never reported in promoters of genes down-regulated in cancer. The concept was later reinforced in small sets of RNA-seq data [9,10]. Overall, these studies retrieved “proliferative” cancer signatures, often enriched in *cell-cycle*, *signaling* and *metabolism* terms. NF-Y is acknowledged as essential for activation of many cell-cycle genes, and the majority, if not all, G2/M genes [5]. Here, the cluster of 1905 genes globally overexpressed in LUSC tumors corresponds, by and large, to the pro-proliferative signature previously identified upon analysis of the 178 tumors by TCGA [26]. Analysis of the four subtypes reinforces that the CCAAT box takes little, if any, part in regulation of subtype-specific signatures, but rather in the core group of overexpressed genes. We find the NF-Y matrix accompanied by two classes of TFBSs, E2Fs and Sp/KLFs, often associated with CCAAT. Indeed, there is robust evidence of colocalization of NF-Y with the respective DNA-binding proteins, E2F1/4, Sp1, Sp2, on active promoters in vivo [14,41,42,43,44]. In particular, the role of overexpressed E2F family members in lung cancer is well documented [45,46,47,48].

Our finding of global NF-YA overexpression, while the HFD subunits are not, lends support to the idea that the former is the regulatory, rate-limiting subunit of the trimer. The original indication came from two systems of post-mitotic cells: unlike their cycling precursors, monocytes and myotubes have little NF-YA, and robust levels of NF-YB [49,50,51]. It is reasonable to think that NF-YA overexpression in tumors will form functional trimers with a potential excess of HFD dimers, switching on, or keeping at high constitutive levels, CCAAT-dependent growth-promoting genes. To settle this question in a definitive way, one would need to assess the relative amounts of the three proteins in nuclei. Unfortunately, the precise calculation of the actual number of endogenous molecules of a single TF in a single nucleus is essentially not possible. 

The existence of two major NF-YA splicing isoforms was documented a long time ago [2], but their respective roles have been unclear ever since. Structurally, NF-YAl contains 28 additional amino acids at the *N*-terminal of the protein, within the large glutamine and hydrophobics-rich trans-activation domain (TAD); these 28 aminoacids have the same relative composition (Gln, Ile/Leu and absence of charged residues) as the surrounding part. On the other hand, both isoforms share the C-terminal subunits-interaction and DNA-binding domains, hence trimerizing and binding CCAAT with identical affinities. In essence, NF-YAl and NF-YAs might impact in differential activation of transcription, rather than selective recognition of distinct CCAAT boxes. Data supporting this view were reported in mESCs [52].

First hints at different biological roles of the isoforms came from stem cell systems, human hematopoietic and mouse embryonic, indicating that NF-YAs is more abundant in “stem”, NF-YAl in differentiated cells [51,52,53,54]. NF-YAl is found relatively high in cells of normal lung (Figure 2B, 2E), but it’s impossible to partition isoforms expression in the many types of cells present in lungs. Tumors samples also contain different cell types, including normal infiltrating immune cells, which are expected to express mainly NF-YAl. Hence, the majority of LUSC show a remarkable increase in NF-YAs, likely to be ascribed to growing epithelial tumor cells within the mass. NF-YAs^high^ expressing tumors are associated with the above-mentioned proliferative signature of CCAAT-dependent genes and, indeed, with the proliferative markers shown in Figure 3. Incidentally, all their promoters are *bona fide* NF-Y targets, with the exception of Ki67.

Another interesting finding was the identification of a group of tumors, showing the opposite isoforms composition, namely low ratios of NF-YAs/NF-YAl. These tend to be NF-YAl^high^, although attempts aimed at purely correlate the expression levels of NF-YAl alone with either prognosis or a particular gene signature yielded negative results (not shown). It is rather the relative ratio of the two isoforms, hence taking into account also NF-YAs levels, that marks this population. The PFI curves of such tumors are very similar to the NF-YAs^high^ cohort, and clearly worse than those of tumors with intermediate isoforms levels. Analysis of the gene expression profiles gave very different, almost opposite results in the two cohorts: metabolism and cell-cycle genes up-regulated in NF-YAs^high^ tumors are rather down-regulated in this cohort, which instead shows up-regulation of a pro-migration, signature. Interestingly, the substantial absence of CCAAT in the promoters of these genes suggest that NF-YAl per se does not directly activates these genes, as NF-YAs does for metabolism and cell-cycle genes, but it might promote them indirectly, by activating yet to be identified TF(s).

This scenario is somewhat reminiscent of what we recently found in breast carcinomas: NF-YAs is also generally overexpressed in these tumors, but we identified a distinct subclass within basal-like tumors, ER^−^/PR^−^/ERBB2^−^, triple negatives, which are NF-YAl^high^ [35]. In this case, we were able to further characterize NF-YAl^high^ tumors, being Claudins^low^, with high levels of EMT markers and low levels of basal Keratins. This BRCA Claudin^low^ cohort is known to be more aggressive and prone to metastasize. In accordance with this, analysis of several BRCA cells lines found an excellent agreement between the relative levels of NF-YA isoforms mRNA and proteins [35]: although the same is likely to happen in LUSC as well, this point was not evaluated here, and will have to be a further line of future experimentation. The partitioning of NF-YA^high^/NF-Yl^low^ in LUSC is less sharp, but we observe the same trend. We therefore hypothesize that NF-YAl is involved in a program of increased expression of mesenchymal genes, not targeted by NF-YAs. The molecular comprehension of the transcriptional mechanisms by which NF-YAl potentially promotes—indirectly—the expression of CCAAT-less pro-migration genes becomes a priority.

Finally, it will also be interesting to verify such findings in the other major type of lung cancer, adenocarcinoma (LUAD): the partitioning of GEO samples described here retrieved 91 LUAD tumors: as organized here, these data will be helpful in determining the relative expression levels of the isoforms and the presence of the CCAAT in overexpressed genes. 

## Supplementary Materials

The following are available online at https://www.mdpi.com/2073-4425/10/11/937/s1, Figure S1: partitioning of GSE81089 data. A. Correlation plot B. PCA. A. Heatmap of signature genes expression across sample classified. Figure S2: splicing of NF-YC. Figure S3: expression of genes hallmarks for each subtype. Figure S4: Global NF-YA expression levels in LUSC subtypes. A. Comparison of relative NF-YA global levels in LUSC subtypes in microarray data. B. Global NF-YA levels in TCGA LUSC subtypes according to RNA-seq data. Figure S5: KOBAS analysis of commonly up-regulated genes in LUSC subtypes. Figure S6: analysis of commonly down-regulated genes in LUSC subtypes. A. Venn diagrams. B. Pscan analysis of promoter sequences of commonly down-regulated genes in all LUSC subtypes. C. KOBAS analysis of commonly down-regulated genes in all LUSC subtypes. Table S1: complete list of LUAD and LUSC samples of GSE81089. Table S2: lists of differentially expressed genes in LUSC tumors vs normal tissues. Table S3: partitioning of LUSC tumors according to the four subtypes. Table S4: lists of differentially expressed genes in LUSC subtypes tumors.

## References

[1] Levine M., Cattoglio C., Tjian R. (2014). Looping back to leap forward: Transcription enters a new era. Cell.

[2] Nardini M., Gnesutta N., Donati G., Gatta R., Forni C., Fossati A., Vonrhein C., Moras D., Romier C., Bolognesi M. (2013). Sequence-specific transcription factor NF-Y displays histone-like DNA binding and H2B-like ubiquitination. Cell.

[3] Li X.Y., Hooft van Huijsduijnen R., Mantovani R., Benoist C., Mathis D. (1992). Intron-exon organization of the NF-Y genes. Tissue-specific splicing modifies an activation domain. J. Biol. Chem..

[4] Ceribelli M., Benatti P., Imbriano C., Mantovani R. (2009). NF-YC complexity is generated by dual promoters and alternative splicing. J. Biol. Chem..

[5] Gurtner A., Manni I., Piaggio G. (2017). NF-Y in cancer: Impact on cell transformation of a gene essential for proliferation. Biochim. Biophys. Acta.

[6] Goodarzi H., Elemento O., Tavazoie S. (2009). Revealing global regulatory perturbations across human cancers. Mol. Cell..

[7] Shi Z., Derow C.K., Zhang B. (2010). Co-expression module analysis reveals biological processes, genomic gain, and regulatory mechanisms associated with breast cancer progression. BMC Syst. Biol..

[8] Gusev Y., Riggins R.B., Bhuvaneshwar K., Gauba R., Sheahan L., Clarke R., Madhavan S. (2013). In silico discovery of mitosis regulation networks associated with early distant metastases in estrogen receptor positive breast cancers. Cancer Inform..

[9] Andrews E., Wang Y., Xia T., Cheng W., Cheng C. (2017). Contextual Refinement of Regulatory Targets Reveals Effects on Breast Cancer Prognosis of the Regulome. PLoS Comput. Biol..

[10] Zuo Z.G., Zhang X.F., Ye X.Z., Zhou Z.H., Wu X.B., Ni S.C., Song H.Y. (2016). Bioinformatic analysis of RNA-seq data unveiled critical genes in rectal adenocarcinoma. Eur. Rev. Med. Pharmacol. Sci..

[11] Dolfini D., Zambelli F., Pavesi G., Mantovani R. (2009). A perspective of promoter architecture from the CCAAT box. Cell Cycle..

[12] Bisikirska B., Bansal M., Shen Y., Teruya-Feldstein J., Chaganti R., Califano A. (2016). Elucidation and Pharmacological Targeting of Novel Molecular Drivers of Follicular Lymphoma Progression. Cancer Res..

[13] Tong Y., Merino D., Nimmervoll B., Gupta K., Wang Y.D., Finkelstein D., Dalton J., Ellison D.W., Ma X., Zhang J. (2015). Cross-Species Genomics Identifies TAF12, NFYC, and RAD54L as Choroid Plexus Carcinoma Oncogenes. Cancer Cell..

[14] Fleming J.D., Pavesi G., Benatti P., Imbriano C., Mantovani R., Struhl K. (2013). NF-Y coassociates with FOS at promoters, enhancers, repetitive elements, and inactive chromatin regions, and is stereo-positioned with growth-controlling transcription factors. Genome Res..

[15] Xie D., Boyle A.P., Wu L., Zhai J., Kawli T., Snyder M. (2013). Dynamic trans-acting factor colocalization in human cells. Cell.

[16] Mamat S., Ikeda J., Tian T., Wang Y., Luo W., Aozasa K., Morii E. (2011). Transcriptional Regulation of Aldehyde Dehydrogenase 1A1 Gene by Alternative Spliced Forms of Nuclear Factor Y in Tumorigenic Population of Endometrial Adenocarcinoma. Genes Cancer.

[17] Cicchillitti L., Corrado G., Carosi M., Dabrowska M.E., Loria R., Falcioni R., Cutillo G., Piaggio G., Vizza E. (2017). Prognostic role of NF-YA splicing isoforms and Lamin A status in low grade endometrial cancer. Oncotarget.

[18] Yang C., Zhao X., Cui N., Liang Y. (2017). Cadherins Associate with Distinct Stem Cell-Related Transcription Factors to Coordinate the Maintenance of Stemness in Triple-Negative Breast Cancer. Stem Cells Int..

[19] Cao B., Zhao Y., Zhang Z., Li H., Xing J., Guo S., Qiu X., Zhang S., Min L., Zhu S. (2018). Gene regulatory network construction identified NFYA as a diffuse subtype-specific prognostic factor in gastric cancer. Int. J. Oncol..

[20] Bie L.Y., Li D., Mu Y., Wang S., Chen B.B., Lyu H.F., Han L.L., Nie C.Y., Yang C.C., Wang L. (2018). Analysis of cyclin E co-expression genes reveals nuclear transcription factor Y subunit α is an oncogene in gastric cancer. Chronic Dis. Transl. Med..

[21] Cui H., Zhang M., Wang Y., Wang Y. (2016). NF-YC in glioma cell proliferation and tumor growth and its role as an independent predictor of patient survival. Neurosci. Lett..

[22] Kottorou A.E., Antonacopoulou A.G., Dimitrakopoulos F.I., Tsamandas A.C., Scopa C.D., Petsas T., Kalofonos H.P. (2012). Altered expression of NFY-C and RORA in colorectal adenocarcinomas. Acta Histochem..

[23] Chen Z., Fillmore C.M., Hammerman P.S., Kim C.F., Wong K.K. (2014). Non-small-cell lung cancers: A heterogeneous set of diseases. Nat. Rev. Cancer.

[24] Relli V., Trerotola M., Guerra E., Alberti S. (2019). Abandoning the Notion of Non-Small Cell Lung Cancer. Trends Mol. Med..

[25] Wilkerson M.D., Yin X., Hoadley K.A., Liu Y., Hayward M.C., Cabanski C.R., Muldrew K., Miller C.R., Randell S.H., Socinski M.A. (2010). Lung squamous cell carcinoma mRNA expression subtypes are reproducible, clinically important, and correspond to normal cell types. Clin. Cancer Res..

[26] Cancer Genome Atlas Network (2012). Comprehensive genomic characterization of squamous cell lung Cancers. Nature.

[27] Mezheyeuski A., Bergsland C.H., Backman M., Djureinovic D., Sjöblom T., Bruun J., Micke P. (2018). Multispectral imaging for quantitative and compartment-specific immune infiltrates reveals distinct immune profiles that classify lung cancer patients. J. Pathol..

[28] Girard L., Rodriguez-Canales J., Behrens C., Thompson D.M., Botros I.W., Tang H., Xie Y., Rekhtman N., Travis W.D., Wistuba I.I. (2016). An Expression Signature as an Aid to the Histologic Classification of Non-Small Cell Lung Cancer. Clin. Cancer Res..

[29] Love M.I., Huber W., Anders S. (2014). Moderated estimation of fold change and dispersion for RNA-seq data with DESeq2. Genome Biol..

[30] Zambelli F., Pesole G., Pavesi G. (2009). Pscan: Finding over-represented transcription factor binding site motifs in sequences from co-regulated or co-expressed genes. Nucleic Acids Res..

[31] Pavesi G., Mereghetti P., Mauri. G., Pesole G. (2004). Weeder Web: Discovery of transcription factor binding sites in a set of sequences from co-regulated genes. Nucleic Acids Res..

[32] Bembom O. (2018). seqLogo: Sequence Logos for DNA Sequence Alignments. R package version 1.48.0. https://pypi.org/project/seqlogo/0.0.1/.

[33] Borcherding N., Bormann N.L., Voigt A.P., Zhang W. (2018). TRGAted: A web tool for survival analysis using protein data in the Cancer Genome Atlas. F1000Research.

[34] Therneau T. (2015). Survival. https://cran.r-project.org/web/packages/survival/citation.html.

[35] Dolfini D., Andrioletti V., Mantovani R. (2019). Overexpression and alternative splicing of NF-YA in breast cancer. Sci. Rep..

[36] Yang D.K., Son C.H., Lee S.K., Choi P.J., Lee K.E., Roh M.S. (2009). Forkhead box M1 expression in pulmonary squamous cell carcinoma: Correlation with clinicopathologic features and its prognostic significance. Hum. Pathol..

[37] Shan L., Zhao M., Lu Y., Ning H., Yang S., Song Y., Chai W., Shi X. (2019). CENPE promotes lung adenocarcinoma proliferation and is directly regulated by FOXM1. Int. J. Oncol..

[38] Zhang C., Min L., Zhang L., Ma Y., Yang Y., Shou C. (2016). Combined analysis identifies six genes correlated with augmented malignancy from non-small cell to small cell lung cancer. Tumour Biol..

[39] Wen P., Chidanguro T., Shi Z., Gu H., Wang N., Wang T., Li Y., Gao J. (2018). Identification of candidate biomarkers and pathways associated with SCLC by bioinformatics analysis. Mol. Med. Rep..

[40] Liu J., Lichtenberg T., Hoadley K.A., Poisson L.M., Lazar A.J., Cherniack A.D., Kovatich A.J., Benz C.C., Levine D.A., Lee A.V. (2018). An Integrated TCGA Pan-Cancer Clinical Data Resource to Drive High-Quality Survival Outcome Analytics. Cell.

[41] Linhart C., Elkon R., Shiloh Y., Shamir R. (2005). Deciphering transcriptional regulatory elements that encode specific cell cycle phasing by comparative genomics analysis. Cell Cycle.

[42] Halperin Y., Linhart C., Ulitsky I., Shamir R. (2009). Allegro: Analyzing expression and sequence in concert to discover regulatory programs. Nucleic Acids Res..

[43] Dolfini D., Zambelli F., Pedrazzoli M., Mantovani R., Pavesi G. (2016). A high definition look at the NF-Y regulome reveals genome-wide associations with selected transcription factors. Nucleic Acids Res..

[44] Wang J., Zhuang J., Iyer S., Lin X., Whitfield T.W., Greven M.C., Pierce B.G., Dong X., Kundaje A., Cheng Y. (2012). Sequence features and chromatin structure around the genomic regions bound by 119 human transcription factors. Genome Res..

[45] Eymin B., Gazzeri S., Brambilla C., Brambilla E. (2001). Distinct pattern of E2F1 expression in human lung tumours: E2F1 is upregulated in small cell lung carcinoma. Oncogene.

[46] Huang C.L., Liu D., Nakano J., Yokomise H., Ueno M., Kadota K., Wada H. (2007). E2F1 overexpression correlates with thymidylate synthase and survivin gene expressions and tumor proliferation in non small-cell lung cancer. Clin. Cancer Res..

[47] Park S.A., Platt J., Lee J.W., López-Giráldez F., Herbst R.S., Koo J.S. (2015). E2F8 as a Novel Therapeutic Target for Lung Cancer. J. Natl. Cancer Inst..

[48] Gao Z., Shi R., Yuan K., Wang Y. (2016). Expression and prognostic value of E2F activators in NSCLC and subtypes: A research based on bioinformatics analysis. Tumour Biol..

[49] Farina A., Manni I., Fontemaggi G., Tiainen M., Cenciarelli C., Bellorini M., Mantovani R., Sacchi A., Piaggio G. (1999). Down-regulation of cyclin B1 gene transcription in terminally differentiated skeletal muscle cells is associated with loss of functional CCAAT-binding NF-Y complex. Oncogene.

[50] Marziali G., Perrotti E., Ilari R., Coccia E.M., Mantovani R., Testa U., Battistini A. (1999). The activity of the CCAAT-box binding factor NF-Y is modulated through the regulated expression of its A subunit during monocyte to macrophage differentiation: Regulation of tissue-specific genes through a ubiquitous transcription factor. Blood.

[51] Gurtner A., Manni I., Fuschi P., Mantovani R., Guadagni F., Sacchi A., Piaggio G. (2003). Requirement for down-regulation of the CCAAT-binding activity of the NF-Y transcription factor during skeletal muscle differentiation. Mol. Biol. Cell.

[52] Dolfini D., Minuzzo M., Pavesi G., Mantovani R. (2012). The short isoform of NF-YA belongs to the embryonic stem cell transcription factor circuitry. Stem Cells.

[53] Zhu J., Zhang Y., Joe G.J., Pompetti R., Emerson S.G. (2005). NF-Ya activates multiple hematopoietic stem cell (HSC) regulatory genes and promotes HSC self-renewal. Proc. Natl. Acad. Sci. USA.

[54] Domashenko A.D., Danet-Desnoyers G., Aron A., Carroll M.P., Emerson S.G. (2010). TAT-mediated transduction of NF-YA peptide induces the ex vivo proliferation and engraftment potential of human hematopoietic progenitor cells. Blood.

