# Supplementary material for: NF-YA Overexpression in Lung Cancer: LUSC
Source: Genes (Basel). 2019 Nov 17;10(11):937. doi: 10.3390/genes10110937 (PMC6895822; doi:10.3390/genes10110937)

Supplementary Figure 1: Partitioning of GSE81089 data. A. Correlation plot B. PCA. C. Heatmap of signature genes expression across samples classified.

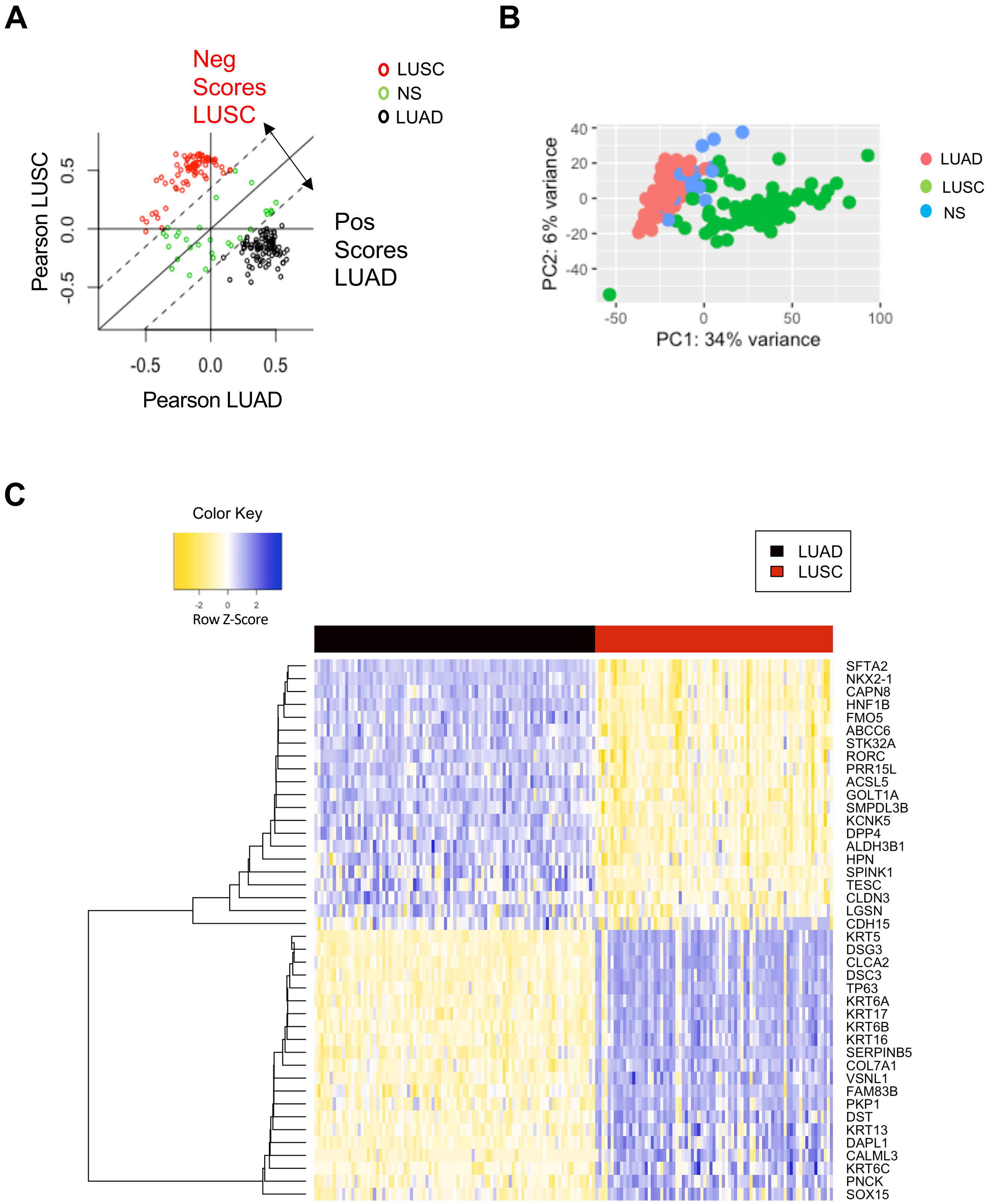

Supplementary Figure 2: Splicings of NF-YC.

NFYC

RefSeq genes	transcript variant	UCSC genes	assembly
NM_001142588	transcript variant 1	uc010ojm.1	hg19
NM_001142588	transcript variant 1	uc001cfx.4	hg19
NM_001142588	transcript variant 1	uc001cge.3	hg19
NM_014223	transcript variant 2	uc009vwd.3	hg19
NM_014223	transcript variant 2	uc001cfy.4	hg19
NM_014223	transcript variant 2	uc001cgb.3	hg19
NM_014223	transcript variant 2	uc001cgd.2	hg18*
NM_001142587	transcript variant 3	uc001cfz.3	hg19
NM_001142589	transcript variant 4	uc010ojn.2	hg19
NM_001142590	transcript variant 5	uc001cgc.3	hg19
NM_001308114	transcript variant 6	-	hg19
NM_001308115	transcript variant 7	-	hg19

NF-YC hg19

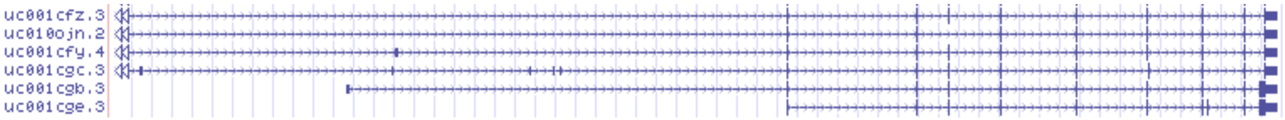

Supplementary Figure 3: Expression of genes hallmarks for each subtype.

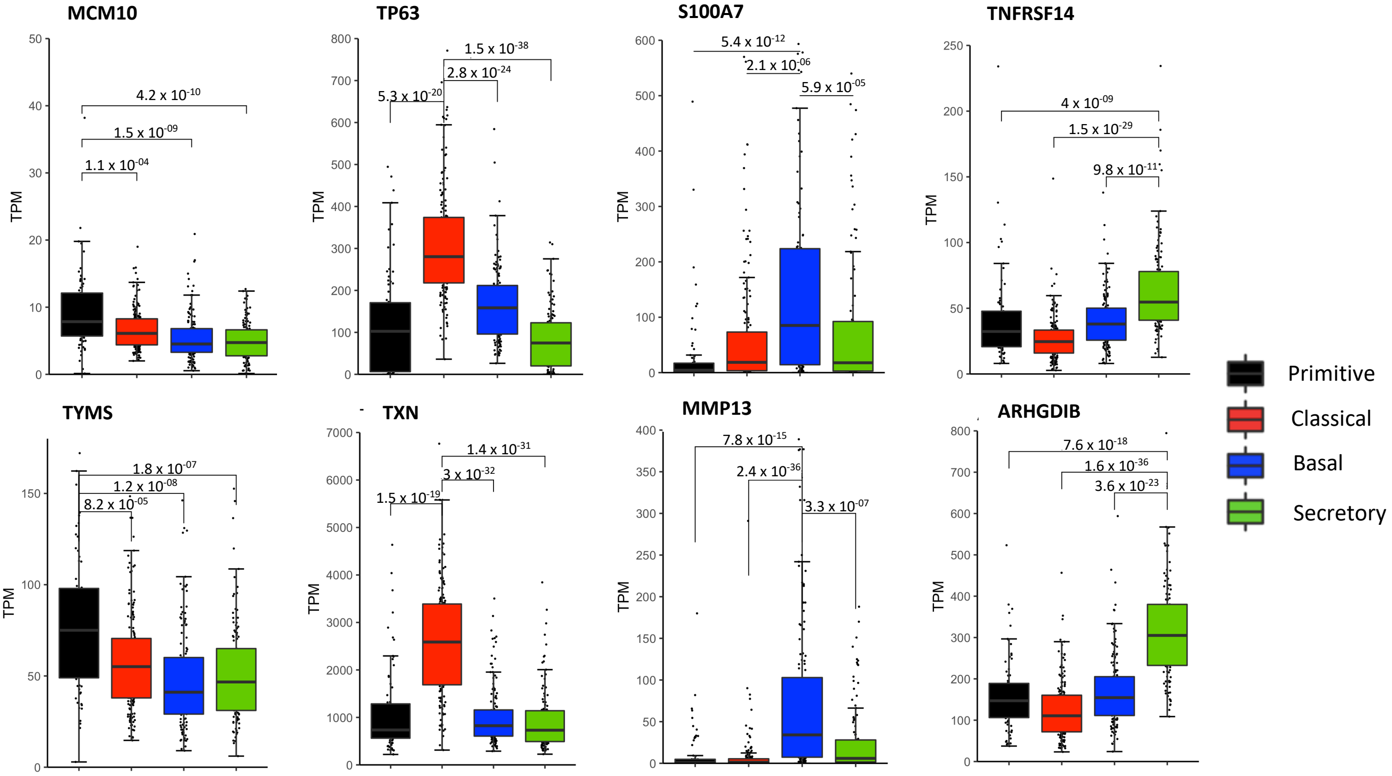

Supplementary Figure 4: Global NF-YA expression levels in LUSC subtypes. A. Comparison of relative NF-YA global levels in LUSC subtypes in microarray data. B. Global NF-YA levels in TCGA LUSC subtypes according to RNA-Seq data.

A

	adj.P.Val	P.Value	t	B	log2FoldChange	SUBTYPE VS OTHERS
NFYA	0.8959	4.95×10^{-01}	-0.695069	-5.10996	-0.7527895	CLASSICAL
NFYA	0.8270491	6.32×10^{-01}	-0.4864997	-5.63337	-0.4043451	SECRETORY
NFYA	1.15×10^{-01}	1.15×10^{-01}	-1.6015297	-4.6244	-0.2803957	BASAL
NFYA	8.89×10^{-01}	7.82×10^{-01}	0.28083	-5.92554	0.23337	PRIMITIVE

B

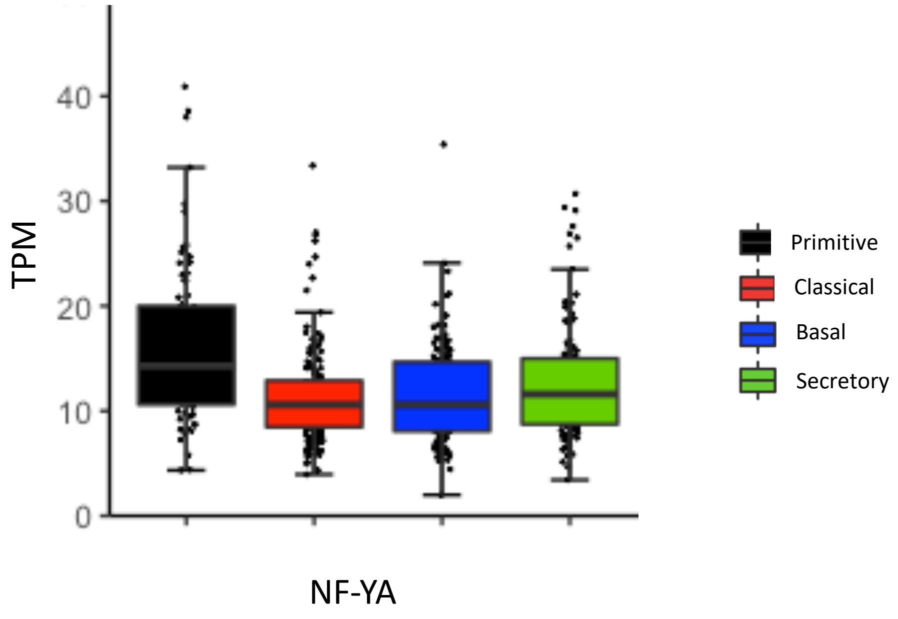

Supplementary Figure 5: KOBAS analysis of commonly up-regulated genes in LUSC subtypes.

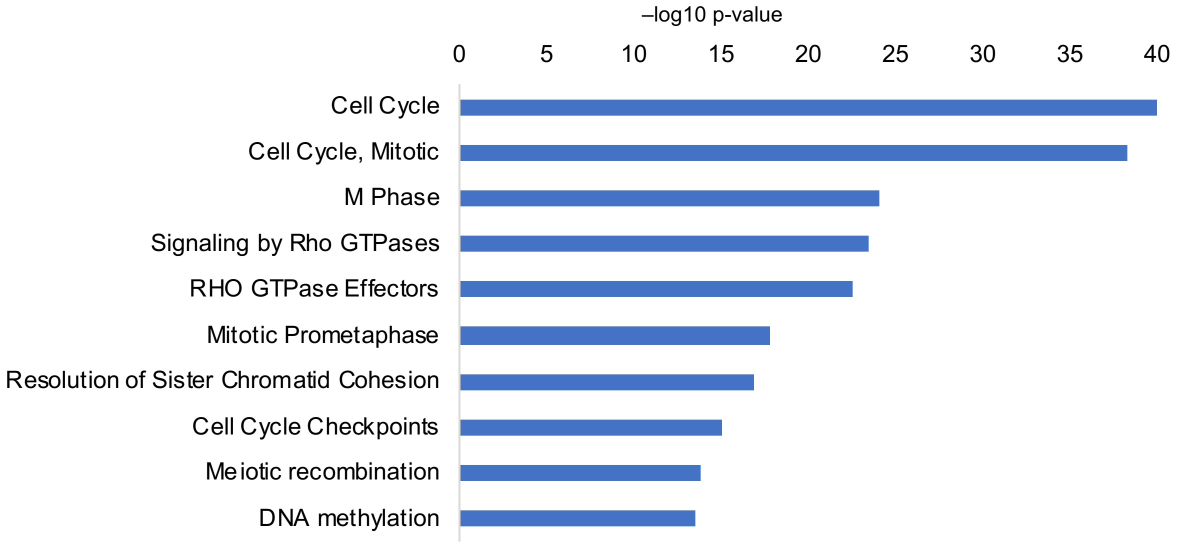

Supplementary Figure 6: Analysis of commonly down-regulated genes in LUSC subtypes. A.Venn diagrams B. Pscan analysis of promoter sequences of commonly downregulated genes in all LUSC subtypes C. KOBAS analysis of commonly downregulated genes in all LUSC subtypes.

A

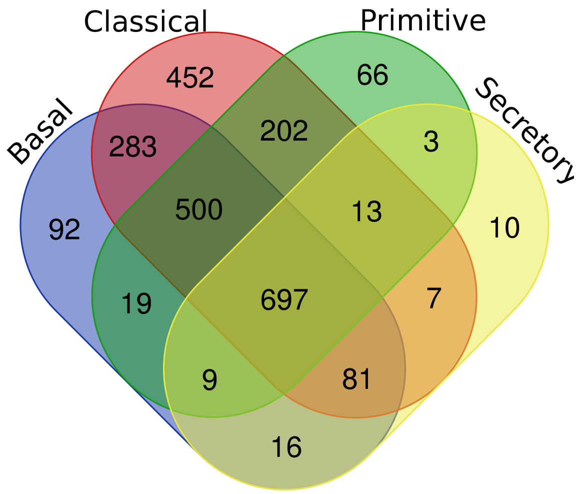

B

TF NAME	P-VALUE
SRF	5.45×10^{-10}
GATA1::TAL1	2.64×10^{-07}
SRF	3.18×10^{-07}
Gata1	3.76×10^{-07}
Tal1::Gata1	9.61×10^{-07}
FOXO6	1.75×10^{-06}
FOXO4	2.86×10^{-06}
FOXL1	3.75×10^{-06}
FOXD2	4.37×10^{-06}
RFX5	5.04×10^{-06}

C

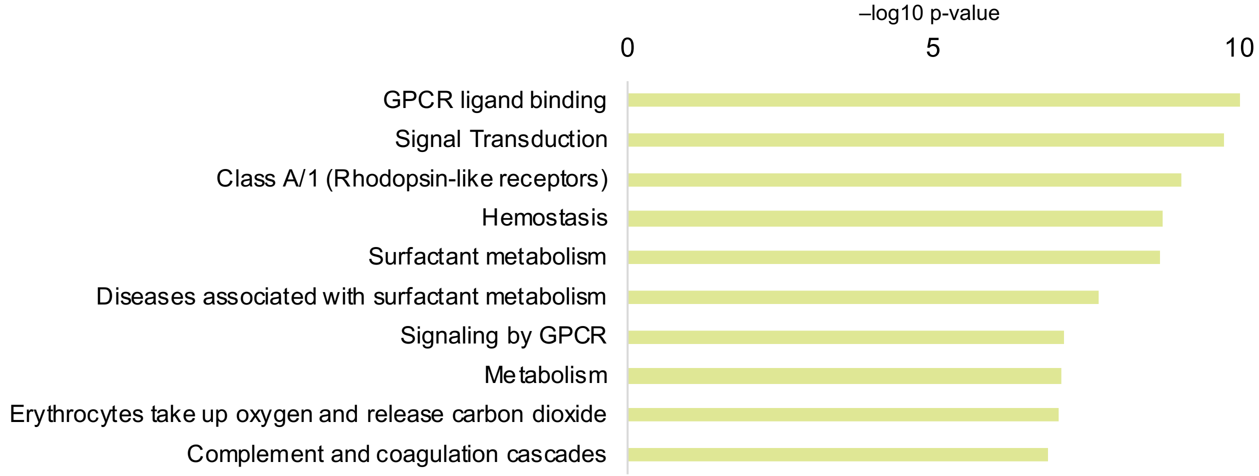

Supplement: Supplementary file 1 [file genes-10-00937-s001.zip › supplementary 3/LUSCSupplementary3.pdf]
